# Predictors of negotiated prices for new drugs in Germany

**DOI:** 10.1007/s10198-020-01201-z

**Published:** 2020-05-25

**Authors:** Afschin Gandjour, Sofia Schüßler, Thomas Hammerschmidt, Charalabos-Markos Dintsios

**Affiliations:** 1 Frankfurt School of Finance and Management, Frankfurt am Main, Germany; 2 Faculty of Applied Health and Social Sciences, Technical University of Applied Sciences Rosenheim, Rosenheim, Germany; 3 Institute for Health Services Research and Health Economics, Centre for Health and Society, Faculty of Medicine, Heinrich-Heine University Düsseldorf, Gebäude 12.49, Moorenstraße 5, 40225 Düsseldorf, Germany

**Keywords:** Innovative medicines, Germany, Pricing, Prediction, Negotiation, AMNOG, I18

## Abstract

**Introduction:**

In Germany, all new, innovative medicines are subject to an early benefit assessment by the German Federal Joint Committee with subsequent price negotiation and optional arbitration. The purpose of this study was to identify drivers of negotiated (including arbitrated) prices of new, non-orphan innovative medicines in Germany.

**Methods:**

The analysis considered all non-orphan drugs that underwent a benefit appraisal between January 2011 and June 2016, and displayed a reimbursement price in the German Drug Directory (Lauer-Taxe^®^) in November 2017. Negotiated annual treatment costs were analyzed with respect to 11 explanatory variables in regression models.

**Results:**

The total sample included 106 non-orphan drugs. The analysis showed a significant and positive association of log-transformed negotiated annual treatment cost of new medicines with log-transformed annual treatment cost of its comparator(s), extent of added benefit, and log-transformed size of the target population. Analyzing the effects of specific endpoints instead of the overall added benefit revealed that the single endpoint with the largest impact on price is adverse events (AEs). Surprisingly, an increase in AEs significantly increased the price. Various subgroup and sensitivity analyses demonstrated the robustness of the results. The adjusted *R* squared for all models was above 80%.

**Conclusions:**

The analysis was able to confirm that variables whose consideration is mandated by law are, in fact, the key drivers of negotiated prices. Somewhat puzzling, the analysis also found an increase in AEs to move prices significantly upward.

**Electronic supplementary material:**

The online version of this article (10.1007/s10198-020-01201-z) contains supplementary material, which is available to authorized users.

## Introduction

In Germany, legislation regulating the reimbursement of new, innovative medicines (new therapeutic entities) within the statutory healthcare system (Arzneimittelmarkt-Neuordnungsgesetz) was introduced on 1 January 2011 [1, 2]. According to this law, new products at the time of launch and for any new indication are subject to an early benefit assessment to determine whether there is sufficient evidence of added medical benefits overall and in particular patient subgroups compared to appropriate therapeutic alternatives. Based on the results of the benefit assessment, manufacturers and representatives of the statutory health insurance (SHI) are expected to agree on an appropriate reimbursement price within 6 months, starting from the completion of the benefit appraisal by the German Federal Joint Committee (Gemeinsamer Bundesausschuss, G-BA). If manufacturers and health insurers cannot agree on the reimbursement price, a final decision on the price will be made by an arbitration board [3]. If one of the parties involved wishes so, the Institute for Quality and Efficiency in Health Care (Institut für Qualität und Wirtschaftlichkeit im Gesundheitswesen, IQWiG) can be commissioned with a formal evaluation of costs and benefits of the product in question.

Based on the German Social Code Book no. 5 (§130b), price negotiations between manufacturers and representatives of the SHI are confidential. Based on section 6 of the framework agreement (Rahmenvereinbarung) [4] pertaining to the same paragraph, the main points to be considered in the negotiations are: (i) the annual costs of the appropriate comparator therapies, as defined by the G-BA, (ii) the extent of the added benefit as a result of the early benefit assessment, expressed by the respective categories together with the uncertainty of the submitted evidence (i.e., the evidence level), (iii) prices of comparable pharmaceuticals within the authorized indication(s) of the assessed drug, and (iv) European prices in the referenced countries adjusted for purchasing power parity and weighted by the respective sales volumes.

Since the framework agreement [4] refers to the same section explicitly to the content of the appraisal of the G-BA, it implies that the number of patients in each subpopulation with a significantly added benefit as part of the target population is a relevant negotiation criterion, as well. Thus, the implemented approach could be well characterized as a mixed-price calculation, using prevalence data to weight the implicit price in each subpopulation. The same approach applies to different indications of a drug.

To what degree these official but also other factors matter for negotiations and arbitration decisions is a matter of ongoing research, with the negotiation process often described as a black box (decisions by the arbitration board are more transparent [3]). Whereas initial investigations have aimed at explaining negotiated price discounts (the difference between launch prices and negotiated prices), more recently attention has shifted to identifying drivers of the percentage markup on prices of comparator treatments (see, e.g., [5–8]). This shift has been partially motivated by the lack of explanatory factors for negotiated discounts and, in particular, the lack of sensitivity of discounts with respect to the size of added health benefits. The more recent studies, using the percentage markup of negotiated prices as the dependent variable, have, in fact, been able to demonstrate a monotonic relationship with the size of added health benefits [6, 7]. Still, they are limited by a small-sample size and a large variance in percentage markups.

The purpose of our study was to identify drivers of negotiated (including arbitrated) prices of new, non-orphan innovative medicines in Germany. Given limitations of prior studies, we did not use the percentage markup itself as a dependent or independent variable, but designated the negotiated price of the new drug as the dependent variable, while using costs of comparators as an explanatory variable (thus following a recently published analysis of non-oncological orphan drug prices in Germany [9]). As will be shown, this model specification is able to achieve a high explanatory power for negotiated prices. To reduce heterogeneity in our sample and avoid some of the particularities around the benefit appraisal of orphan drugs in Germany (see below for details), we exclusively focused on non-orphan drugs.

## Methods

Our analysis considered all non-orphan drugs that underwent an appraisal by the G-BA between January 2011 and June 2016, and displayed a reimbursement price in the German Drug Directory (Lauer-Taxe^®^) [10] at the time of our analysis (November 2017). This included drugs for which a decision on the reimbursement price was made by the arbitration board. We included drugs that were withdrawn from the German market, but had a publicly available price resulting from negotiation or arbitration. This way, the model contained all available information on drivers of negotiated prices regardless of the manufacturers’ reaction in terms of market withdrawal. We included drugs without additional benefit for which a reimbursement price was negotiated and had to be below the price of the appropriate comparators. However, if adequate therapeutic reference price groups exist, drugs with no additional benefit can be included in those groups and a price is not negotiated. Those drugs were excluded from our analysis. Moreover, we excluded orphan drugs as designated by the European Commission as they do not possess an official comparator set by the G-BA and, therefore, do not permit considering costs of official comparators in the regression equations. Yet, orphan drugs that have passed a €50-million ambulatory budget impact are still included, because they need to undergo a regular assessment and, hence, do possess an official comparator set by the G-BA.

Specifically, we analyzed negotiated annual treatment costs with respect to 11 explanatory variables: (i) annual treatment costs of appropriate comparator(s); (ii) extent/certainty of added therapeutic benefit; (iii) size of target population; (iv) categorization of the comparator(s) as generic, patent-protected, or both; (v) prevalence of the underlying disease; effect on (vi) mortality, (vii) morbidity, (viii) quality of life, and (ix) adverse events (AEs); (x) mortality of the underlying disease; and (xi) therapeutic area. Table 1 presents means, medians, and ranges. We explain the rationale for the choice of these variables in the following sections. Of note, we did not include prices of comparable pharmaceuticals and European reference prices, both of which are official criteria for determining reimbursement prices. For comparable pharmaceuticals, the reason is that they are not explicitly stated and subject to a negotiation themselves. Similarly, European reference prices are not publicly disclosed (but known to the negotiating parties) due to confidential discounts off the official list prices.

**Table 1:** Summary statistics (*n* = 106 drugs)

	Mean	Median	Range
Extent/certainty of added benefit			
Major (%)	1.9	–	–
Considerable (%)	31.1	–	–
Minor (%)	17.0	–	–
Nonquantifiable (%)	2.8	–	–
None (%)	47.2	–	–
De Millas score	3.2	3	0–11
Effect on mortality (% yes)	22.6	–	–
Effect on morbidity (% yes)	42.5	–	–
Effect on quality of life (% yes)	15.1	–	–
Effect on adverse events (% yes)	26.4	–	–
Annual mortality of disease (%)	11.5	0.46	0–88.0
Annual treatment cost^a^ (€)	37,308	12,408	4–468,149
Comparator treatment cost (€)	30,632	8994	12–521,729
Rebate (%)	28	24	0–69
Type of comparator			
Generic (%)	27.4	–	–
Patent-protected (%)	16.0	–	–
Mixed (%)	56.6	–	–
Prevalence of underlying disease	1,322,051	237,590	3552–7,559,770
Number of patients in target population	375,331	40,331	73–7,421,997
Therapeutic area			
Oncology (%)	29	–	–
Infectious diseases (%)	17	–	–
Metabolic (%)	15	–	–
Neurology (%)	8	–	–
Respiratory (%)	7	–	–
Cardiovascular (%)	5	–	–
Hematology (%)	3	–	–
Other (%)	17	–	–

^a^Based on negotiated prices

### Annual treatment costs of new drugs

For the calculation of annual treatment costs of new drugs, we excluded costs of drug administration, monitoring, etc. as the latter are not negotiated. Treatment costs of existing drugs with which the new drug is provided in combination, e.g., in patients with diabetes mellitus or human immunodeficiency virus, were not taken into account either for the same reason. Information on annual treatment costs was obtained from the official resolution documents issued by the G-BA before the price negotiation between a manufacturer and the National Association of SHI Funds. We considered the highest dose for the largest pack size. If unavailable from this source, we retrieved the information from IQWiG’s assessment reports. In cases where several dosing regimens were reported (e.g., on the basis of age or weight), we took the average of the upper and lower bounds of annual treatment costs. In a conservative scenario, we used upper bounds only.

To arrive at the annual treatment cost after discounting, we obtained the difference between the negotiated reimbursement price and the list price from the Lauer-Taxe^®^ [10], divided it by the list price, and applied this percentage to the annual treatment cost before negotiation.

### Annual treatment costs of comparator(s)

In case, several comparators were available, we calculated an average annual treatment cost based on the highest and lowest estimate. In a sensitivity analysis, we tested the upper and lower bound costs. In case, several subgroups were available, average annual treatment costs of comparators in each subgroup were calculated and weighted by the corresponding population size. If information on the latter was not available, we applied an equal weight to each subgroup.

For ‘best supportive care’ (BSC) official documents lacked information on the annual treatment cost. BSC was used as a comparator for seven new medicines. In five of these instances, BSC was used add-on to the new medicine as well. In either case, we applied an estimate of €10,000 in the base case. We derived this estimate from the area of oncology where BSC is often used as an official comparator and typically includes pain medications such as opioids as well as chemotherapy. The daily cost of opioids in Germany is €5.26 ([11], p. 249, Tab. 9.1) or €1920 per year. In cancer patients, strong opioids may be prescribed, however, thus resulting in above-average costs. Patients may receive other pain medications in addition. The daily cost of oncological drugs (excluding protein kinase inhibitors and monoclonal antibodies) is €12.56 or €4585 per year ([11], p. 600, Tab. 37.1). Again, this cost may represent an underestimate as it only refers to a single-agent therapy but not to chemotherapy combination regimens, which are the likely mainstay of treatment for many oncological diseases. To account for the uncertainty of the cost of BSC, we varied it in a sensitivity analysis.

If the G-BA did not provide a specific comparator or a list of specific comparators, we classified the comparator (e.g., ‘best supportive care’) as generic. We reasoned that if the comparator included a patented drug, the latter most likely would have been designated as a specific comparator.

### Extent/certainty of benefit

On the basis of the early benefit appraisal by the G-BA, we used a categorical variable to distinguish between no, ‘non-quantifiable’, ‘minor’, ‘considerable’, and ‘major’ additional benefit. To this end, we used information from the official G-BA resolution documents. In the base-case analysis, we only considered the extent of added benefit. In a sensitivity analysis, we also considered the certainty of benefit as stated in the official G-BA resolution documents. To this end, we used a score published by de Millas et al. [12], which assigns numerical values to combinations of size and certainty of benefit.

When benefit appraisals of several subgroups/subpopulations from the same drug were available, we took data from the subgroup/subpopulation with the largest extent of an added benefit. We assumed that the latter subgroup/subpopulation would exert the largest effect on price. In case, new indications for an existing product were appraised, we took data from the first appraisal. This was based on the observation that the impact of an additional appraisal on the price is rather small [13], i.e., the first appraisal is usually the key driver of the price.

### Size of target population

As an official driver of costs, we included the size of the population expected to have an indication for the drug in the German SHI system. The size of the population with an indication is supposed to be smaller than the total prevalence, because it takes into consideration, among others, contraindications, age restrictions, privately insured patients, and lack of access to treatment, for example, because patients may not be detected. Preferably, we collected information on the size of the target population from G-BA resolution documents. Otherwise, we took this information from IQWiG’s assessment reports or the manufacturers’ value dossiers. In case only ranges were published, we took the average of the upper and lower bounds.

For drugs with an indication for more than one disease, we determined the sum of target population sizes.

### Prevalence

We collected information on disease prevalence from IQWiG’s assessment reports or the manufacturers’ value dossiers. If no such information was available (in the case of regadenoson), we applied the size of the target population. The assumption was that the National Association of SHI Funds had no other information at its disposal either. For drugs with an indication for more than one disease, we determined the sum of prevalence rates.

### Effect on mortality

On the basis of the G-BA resolution documents, we used a dummy variable to categorize mortality changes either granting an added benefit or not.

### Effect on morbidity

On the basis of the G-BA resolution documents, we used a dummy variable to categorize the presence of an added benefit based on morbidity.

### Effect on quality of life

On the basis of the G-BA resolution documents, we used a dummy variable to categorize the presence of an added benefit based on quality of life.

### Effect on adverse events

On the basis of the G-BA resolution documents, we used two dummy variables to categorize either the presence of an added benefit or the presence of less benefit/harm based on AEs.

### Annual mortality rate by disease

To capture severity of illness, we calculated an annual mortality rate among diseased individuals based on information on the mortality of each disease provided by the German Federal Statistical Office. As a consideration of severity of disease is already mandated for the benefit assessment by law (§ 5 AM-NutzenV) [14], its consideration in price negotiation or arbitration would amount to double counting.

### Therapeutic areas

We chose the two therapeutic areas with the highest number of drugs available (oncology and infectious diseases). We did not include other therapeutic areas due to the small sample of drugs in each of these areas and the risk of model overfitting.

## Data analysis

We used the method of ordinary least squares (OLS) for estimating the parameters in a linear regression model. All independent variables were continuous except for type of comparator, type of therapeutic area, and extent/probability of added therapeutic benefit, which were discrete. We conducted the Shapiro–Wilk test to determine whether or not (log transformed) continuous variables were normally distributed. We considered *p* values of < 0.05 to be statistically significant. To detect multicollinearity among the continuous explanatory variables, we constructed a correlation matrix. To test the independence between two categorical variables, we used the Pearson’s chi-square contingency table test. In addition, we calculated variance inflation factors (VIFs), which measure how much the variance of the estimated regression coefficients is inflated [15]. To deal with potential heteroscedasticity, we estimated the regressions with robust standard errors.

In the first set of regressions, we only included independent variables that are mandated by law to exert an influence over the results of the price negotiation (based on section 6 of the framework agreement [4] according to § 130b of the Social Code Book no. 5, which includes a reference to § 35a): annual treatment costs of appropriate comparator(s); extent/certainty of added therapeutic benefit; and size of target population. Next, we added variables that are not prescribed by law, but may play a role unofficially. We conducted separate analyses for pricing through negotiation and arbitration.

As the primary goal of our regression was to explain and not to predict annual treatment costs, we did not develop a parsimonious model based on stepwise elimination. All analyses were performed using Stata version 11.0 (Stata Corporation, College Station, TX, USA).

## Results

### Variables

The total sample included 106 non-orphan drugs. The Chi-square independence test yielded the following significant associations (*p* < 0.05) between two categorical variables: categorization of the comparator and type of therapeutic area (in particular, between categorization of the comparator as a generic drug and metabolic disease); extent of added benefit and effect on mortality (specifically, between increase in added benefit and effect on mortality); type of therapeutic area (infectious/oncological disease); and effect on mortality.

Because the Shapiro–Wilk test showed a violation of normality for annual treatment cost of the new medicine and its comparator(s) as well as for prevalence of the underlying disease and number of patients in the target population, we log-transformed these variables. The resulting log–log models allowed us to interpret estimated parameters as elasticities.

Correlation matrix and two-way contingency tables indicated multicollinearity between log-transformed average, highest and lowest annual treatment costs of comparator(s); benefit classification with and without consideration of the certainty of benefit; extent of added benefit and effect on mortality, morbidity, quality of life, and AEs; and log-transformed total prevalence and log-transformed size of target population. Therefore, we did not include these variables in one model and instead kept them in separate models.

In the regressions that are reported in the following, all VIFs were well below the critical value of 10, indicating that multicollinearity between independent variables was not a concern.

### Regression models

We found a significant and positive association of log-transformed negotiated annual treatment cost of the new medicine with log-transformed annual treatment cost of its comparator(s), extent of added benefit, and log-transformed size of the target population (Table 2, model 1). Increasing the extent of added benefit by one level increased the cost of the new medicine by 23%. On the other hand, size of the target population had a significantly negative relationship with annual treatment cost of the new medicine: Doubling the size of the target population led to a 13% decrease in annual treatment cost. A similar result was obtained when including the certainty of benefit in the benefit classification. Increasing the extent of added benefit by one level increased the cost of the new medicine by 38%, while increasing the certainty of benefit from hint to indication or from indication to proof increased the cost of the new medicine by 13%.

**Table 2:** Effects of predictor variables on the logarithm of the negotiated price of non-orphan drugs

	(1)	(2)	(3)	(4)
Log cost of comparator	0.7508069*** (0.0559953)	0.7637213 *** (0.0601955)	0.7836088*** (0.0635719)	0.8005568*** (0.0651212)
Log target pop	− 0.1318565*** (0.0468315)	− 0.1145453** (0.0527203)	− 0.0857794 (0.0548493)	− 0.0950808 (0.0612482)
Added benefit	0.2264448*** (0.0691019)		0.1991282** (0.083959)	
Effect on mortality		0.4592845** (0.2215727)		0.4015837* (0.2099597)
Effect on morbidity		0.159058 (0.2330356)		0.1715455 (0.2558061)
Effect on QoL		− 0.1752775 (0.2981547)		− 0.219479 (0.2915866)
Reduction of AEs		0.073593 (0.2075705)		0.0713919 (0.1968346)
Increase in AEs		0.6838544*** (0.2349997)		0.6567439*** (0.2257846)
Type of comparator			− 0.2110681 (0.1533157)	− 0.2564514 (0.1675672)
Annual mortality			− 0.1704651 (0.3990701)	− 0.2069172 (0.4069993)
Oncology			0.3991902 (0.3672411)	0.168975 (0.318673)
Infectious diseases			− 0.0000717 (0.2943645)	0.1678752 (0.2610417)
Observations	106	106	106	106
Adjusted* R *squared	86%	87%	87%	87%
Log cost of comparator	0.7508069*** (0.0559953)	0.7637213 *** (0.0601955)	0.7836088*** (0.0635719)	0.8005568*** (0.0651212)
Log target pop	− 0.1318565*** (0.0468315)	− 0.1145453** (0.0527203)	− 0.0857794 (0.0548493)	− 0.0950808 (0.0612482)
Added benefit	0.2264448*** (0.0691019)		0.1991282** (0.083959)	
Effect on mortality		0.4592845**0.2215727)		0.4015837* (0.2099597)
Effect on morbidity		0.159058 (0.2330356)		0.1715455 (0.2558061)
Effect on QoL		− 0.1752775 (0.2981547)		− 0.219479 (0.2915866)
Reduction of AEs		0.073593 (0.2075705)		0.0713919 (0.1968346)
Increase in AEs		0.6838544*** (0.2349997)		0.6567439*** (0.2257846)
Type of comparator			− 0.2110681 (0.1533157)	− 0.2564514 (0.1675672)
Annual mortality			− 0.1704651 (0.3990701)	− 0.2069172 (0.4069993)
Oncology			0.3991902 (0.3672411)	0.168975 (0.318673)
Infectious diseases			− 0.0000717 (0.2943645)	0.1678752 (0.2610417)
Observations	106	106	106	106
Adjusted R squared	86%	87%	87%	87%

Standard errors robust against heteroscedasticity are reported in parentheses

*Pop* population, *QoL* quality of life, *AE* adverse event

***p < 0.01, **p < 0.05, *p < 0.10

Subgroup analyses by type of comparator (generic/patent-protected/mixed) did not reveal a significant relationship between extent of added benefit and log-transformed annual treatment cost of the new medicine.

Analyzing the effects of specific endpoints instead of the overall added benefit revealed that the single endpoint with the largest impact on price was AEs (Table 2, model 2). Surprisingly, an increase in AEs significantly increased the price. The sample of drugs showing an increase in AEs included the following 17 drugs: aflibercept, boceprevir, cabazitaxel, cobimetinib, crizotinib, dulaglutide, eribulin, fingolimod, ipilimumab, ramucirumab, ruxolitinib, sacubitril/valsartan, telaprevir, tiotropium/olodaterol, vandetanib, vemurafenib, and vismodegib. The significance of the relationship was insensitive to the assumed annual treatment cost of BSC, which served as a comparator of some of the drugs (the cost was varied between €5000 and €20,000). While an increase in AEs was always counterbalanced by an improvement in other endpoints, it is important to remember that the price increase due to increased AEs cannot be explained by other endpoints, because they were controlled for in the model. We will take up this point in the Discussion. A subgroup analysis that defined an increase in AEs only by serious or Common Terminology Criteria for Adverse Events (CTCAEs) grade ≥ 3 AEs still revealed a significant and positive coefficient (*p* = 0.024). An additional subgroup analysis by type of comparator did not reveal a significant impact of any endpoint including total AEs.

Adding therapeutic areas, indication-specific mortality rate, and type of comparator to the independent variables listed in Table 2, model 1, did not lead to changes in the direction and magnitude of predictors (Table 2, model 3). Among the variables added, type of comparator had the strongest impact on price, although it did not reach statistical significance. When adding therapeutic areas, indication-specific mortality rate, and type of comparator to the independent variables listed in Table 2, model 2, the statistical significance of a positive price effect of an increase in AEs persisted (Table 2, model 4). The Online Appendix shows the Stata commands for models 2 and 4. In addition, testing interactions between an increase in AEs and other variables (each in a separate model) did not reveal statistically significant results. To avoid overfitting and multicollinearity due to the small-sample size, we did not conduct subgroup analyses by type of comparator.

When examining the set of drugs with pricing through negotiation (*n* = 82) based on the set of official pricing variables, the adjusted R squared was 84%. While the extent of added benefit was not significant at the 5% level (*p* = 0.055), analyzing the set of specific endpoints instead of an overall added benefit confirmed the statistically significant impact of an increase in AEs on price (*p* = 0.037).

When analyzing the set of drugs whose reimbursement price was determined through arbitration (*n* = 24) based on the official pricing variables, the adjusted *R* squared was higher than for negotiated prices (91%). This suggests that the arbitration board considers the official pricing variables to a larger extent than the National Association of SHI Funds. The extent of added benefit and the log-transformed annual treatment cost of comparators were significant determinants of prices set through arbitration. Analyzing the set of specific endpoints revealed that an increase in side effects did not have a significant positive impact on price (*p* = 0.114). On the contrary, a decrease in AEs was significantly associated with higher prices (*p* = 0.023).

## Discussion

Our models were able to achieve a high explanatory power, with just 14% of the variance in negotiated reimbursement prices of new, innovative medicines remaining unexplained in the complete sample. Our analysis is able to confirm empirically that variables whose consideration are mandated by law (i.e., costs of comparators, extent of added health benefit, and size of the target population) are, in fact, the key drivers of negotiated prices. Somewhat puzzling, we also found an increase in AEs to move prices significantly upward despite controlling for the (positive) effects of other clinical endpoints, in particular a reduction in mortality. This means that the price-enhancing effect of an increase in AEs goes beyond the positive impact of other endpoints. Still, an increase in harm is always counterbalanced by an improvement in other endpoints. Hence, it is possible to have less benefit in terms of AEs, but still obtain an overall added benefit. We thus conclude that the statistical analysis is consistent in itself and that the price-enhancing effect of AEs does not appear to be the result of a statistical artefact. We do not expect the direction of findings to change with a larger sample size (i.e., inclusion of more recently launched drugs), which would increase the power to detect statistical significance for other endpoints such as mortality (which is statistically significant at the 10% level already). We expect that increasing the power would likewise lead to a smaller *p* value for AEs.

The paradoxical finding of a price-enhancing effect of AEs can be potentially explained in two ways. One is that the increase in AEs may be correlated with price-increasing omitted variables, which were not included in our analysis. Candidate variables are discussed below. The other, more troubling explanation is that our finding reflects a real phenomenon, which is irrational or inconsistent decision-making on behalf of payers. In fact, as the *R* squared values found in our analysis are quite high, relatively little room exists for omitted explanatory variables, rendering inconsistent decision-making the more likely explanation. Arbitration decisions, which show a significant association in the expected direction, i.e., between fewer AEs and higher prices, appear more rational. The finding of a higher proportion of arbitrated than negotiated prices explained supports the hypothesis of a quasi-algorithmic approach by the arbitration board [3] and, thus, perhaps adds face validity to our analysis.

While the purpose of our regression models was explanatory in the first place, it is, nevertheless, possible to use them for the purpose of predicting negotiated prices as well (e.g., from the position of manufacturers). In that case, model parsimony becomes an important objective. As model 1 in Table 2 yields the same adjusted R squared as the others but with fewer variables, it presents the best predictive model.

### Limitations

As our analysis did not consider the results of re-appraisals of added benefit and budget impact of drugs after June 2016, but included the resulting price discounts until November 2017, inconsistency may have resulted in these instances. Given the relatively long average time to re-appraisal of 33 months [8], we do not expect a significant bias, however.

In case, several comparators were available, we calculated the average of the highest and lowest annual treatment cost. In cases where information on the size of subgroups was unavailable, we assumed equal weights for each subgroup. Yet, in price negotiations or arbitrations, equal weights may not have been used.

Price negotiations may also consider a number of factors that were not included in our analysis and may explain the remaining variance in negotiated prices. Officially, negotiations and arbitration board decisions also need to be informed by European reference prices and costs of drugs within the same therapeutic area that are not official clinical comparators set by the G-BA. Unofficial factors may also play a role and potentially include savings from avoided hospitalizations or other treatments, portfolio deals where prices of several drugs of a company’s portfolio are negotiated simultaneously, and R&D costs.

While costs of BSC may have contributed to the high correlation of costs of comparators and negotiated prices in cases where new medicines were used add-on to BSC (due to a potential price pressure), cases where BSC was not part of a combination regime were not captured by a separate variable due to small-sample size (*n* = 2). Still, as our sensitivity analysis using a lower estimate of the costs of BSC showed no significant impact, an impact of costs of BSC on negotiated prices was at least indirectly excluded.

Our classification of the extent of added benefits as well as the score published by de Millas et al. [12], which was used in the sensitivity analysis, are limited by categorically prioritizing minor benefit above unquantifiable benefit.

Nevertheless, the high *R* squared values of our analysis are reassuring in the sense that our variable selection and specifications are largely consistent with real-world decision-making and that the limitations listed above do not seem to carry a critical weight in explaining negotiated prices.

## Conclusion

Our analysis is able to confirm empirically that variables whose consideration are mandated by law are, in fact, the key drivers of negotiated prices. Somewhat puzzling, we also found an increase in AEs to move prices significantly upward. While we cannot rule out an omitted variable bias, the high explanatory power of our models suggests this bias to be rather small.

## Electronic supplementary material

Below is the link to the electronic supplementary material.

Supplementary file1 (DOCX 27 kb)
