# Supplementary material for: Predictors of negotiated prices for new drugs in Germany
Source: Eur J Health Econ. 2020 May 25;21(7):1049–57. doi: 10.1007/s10198-020-01201-z (PMC7423852; doi:10.1007/s10198-020-01201-z)
Supplement: Supplementary file 1 — Supplementary file1 (DOCX 27 kb) [file 10198_2020_1201_MOESM1_ESM.docx]

Appendix

Stata commands:

Table 2, model 2

reg log_cost_drug log_cost_comparator log_target_population effect_mortality

effect_morbidity effect_QoL reduction_AEs increase_AEs, r

Table 2, model 4

reg log_cost_drug log_cost_comparator log_target_population effect_mortality

effect_morbidity effect_QoL reduction_AEs increase_AEs type_comparator

mortality_annual TA_oncology TA_infectious_D, r
